# Bone marrow niches in myeloid neoplasms

**DOI:** 10.1111/pin.12870

**Published:** 2019-11-10

**Authors:** Masanobu Kitagawa, Morito Kurata, Iichiroh Onishi, Kouhei Yamamoto

**Affiliations:** ^1^ Department of Comprehensive Pathology, Graduate School of Medical and Dental Sciences Tokyo Medical and Dental University Tokyo Japan

**Keywords:** hematopoietic stem cell niche, MDS, microenvironment, myeloid neoplasms

## Abstract

Pathological phenotypes of myeloid neoplasms are closely related to genetic/chromosomal abnormalities of neoplastic cells whereas the bone marrow microenvironment, including stromal elements and hematopoietic stem cell niche cells, have a great influence on the differentiation/proliferation of both hematopoietic and neoplastic cells. The pathology of myeloid neoplasms might be generated through the interaction of hematopoietic (stem) cells and stromal cells. The present study aims to provide the morphological/functional aspects of the bone marrow environment in myeloid neoplasms. Among the myeloid neoplasms, myelodysplastic syndromes (MDS) exhibit significant and complex interactions between neoplastic cells and stromal cells. Hematopoietic cells in MDS are greatly influenced by macrophages/niche cells via several signaling pathways. As such, the pathological significance of cell proliferation, cell apoptosis, and anti‐apoptosis signals in the bone marrow of myeloid neoplasms, especially MDS bone marrow, will be discussed.

**Abbreviations**
AML‐MRC: AML with myelodysplasia related change
ET: essential thrombocythemia
MCM2: minichromosome maintenance 2
MDS: myelodysplastic syndromes
MDS→OL: MDS cases that later transformed to overt leukemia
MPN: myeloproliferative neoplasms
MVD: microvascular density
pMF: primary myelofibrosis
PV: polycythemia vera

## INTRODUCTION

It is well known that some myeloid neoplasm cases harbor different kinds of genetic mutations/chromosome abnormalities in both hematopoietic and bone marrow stromal cells. Although the genome‐wide analysis of neoplastic cells in myeloid neoplasms has revealed numerous genetic alterations,^1^, ^2^, ^3^, ^4^, ^5^ experiments using genetically modified animal models have shown that only the alterations of bone marrow stromal cells (niche cells) result in the pathologic evolution of myelodysplastic syndromes (MDS) and myeloproliferative neoplasms (MPN) in host animals.^6^, ^7^ Also in human samples, mesenchymal stromal cells of MDS/AML bone marrow have been shown to harbor chromosomal abnormalities such as translocation, deletion and inversion that were not observed in hematopoietic blasts.^8^ Thus, the significance of the genetic alterations of hematopoietic cells themselves/hematopoietic cell niche is still controversial.^8^, ^9^, ^10^, ^11^

In previous studies, we have shown that during MDS, bone marrow cells exhibit frequent apoptosis in the bone marrow through the interactions of hematopoietic cells with the bone marrow stromal cells.^12^, ^13^, ^14^, ^15^ As such, we would like to propose novel aspects of myeloid neoplasms by analyzing the pathology of neoplastic cells/stromal niche cells.

## BONE MARROW MICROENVIRONMENT OF MYELOID NEOPLASMS

### Types of cells comprising the bone marrow microenvironment

**Figure 1:**
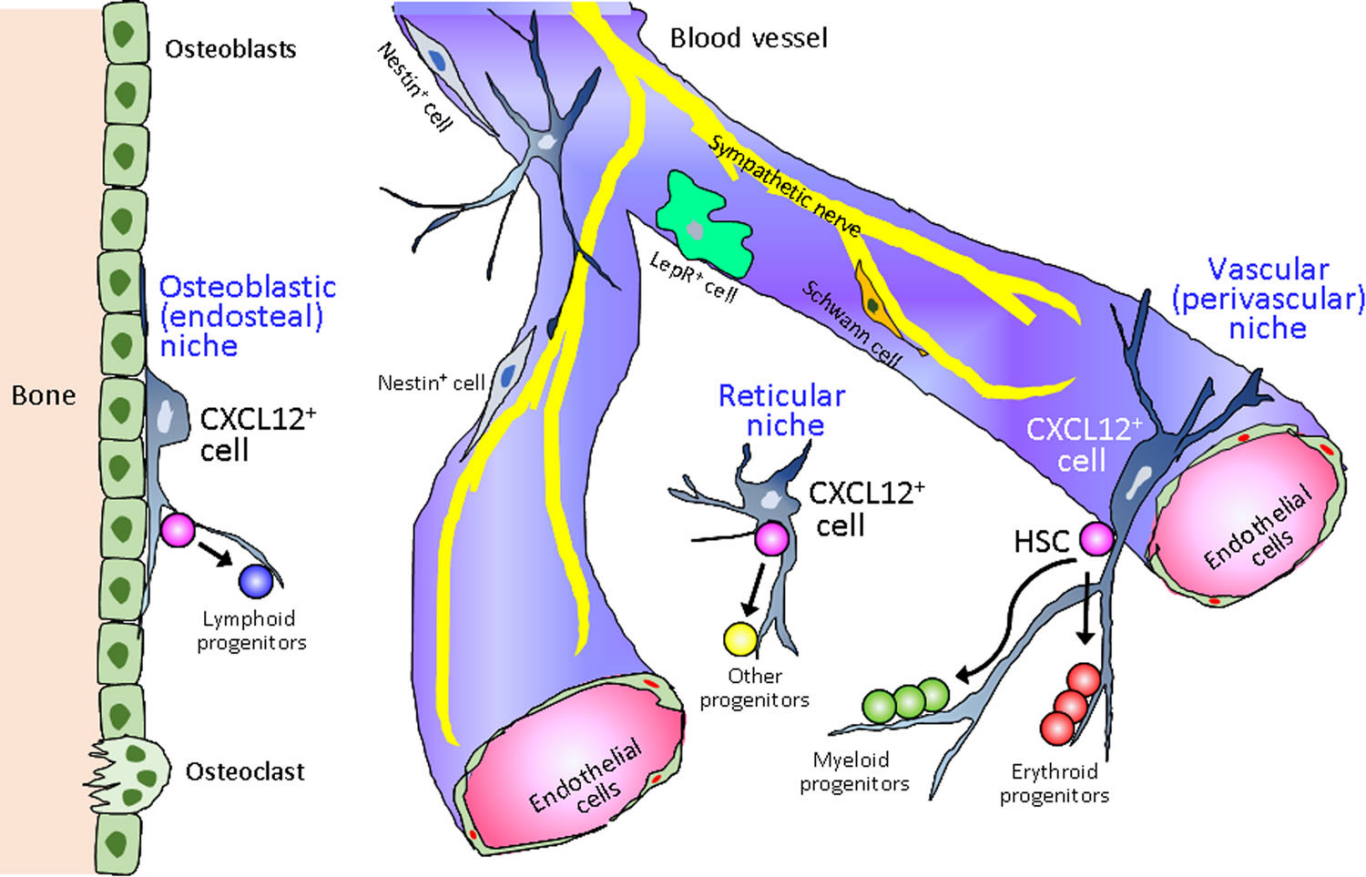
Candidate cells constituting the bone marrow niche (hematopoietic stem cell niche). There are several types of cells that could be considered as niche constituting cells in the bone marrow, including CXCL12‐positive reticular cells, Nestin‐positive cells, Leptin receptor‐positive cells, and Schwann cells. Among these candidate cells, CXCL12‐positive cells are the most frequently observed in the bone marrow. CXCL12‐positive cells are located among the hematopoietic cells in the perivascular area and capillaries or sinusoids (perivascular niche), in the parenchyma of the bone marrow (reticular niche), as well as in the endosteal region (osteoblastic niche). Niche cells seem to have specific roles in the differentiation of hematopoietic stem cells (HSC) to erythroid, myeloid or lymphoid progenitor cells.

What types of cells constitute the bone marrow microenvironment as well as the so‐called hematopoietic stem cell niche? Many types of cells have been nominated as candidates for niche‐forming cells, being mainly identified by using mouse models.^16^ These include CXCL12‐associated reticular cells (CAR cells), nestin‐positive cells, leptin receptor‐expressing cells, GFAP‐positive cells and many others (Fig. 1).^17^, ^18^, ^19^ Among these cells, the CXCL12‐positive cells are the most frequently observed cells in the bone marrow. Thus, we subsequently focused on CXCL12‐positive cells as the representative candidate for niche‐forming cells of the bone marrow.^20^, ^21^ The CXCL12‐positive cells of the human bone marrow exist in the perivascular space at the center of the hematopoietic cell foci, in the spaces surrounding adipose cells, in peri‐osteoblastic spaces, and so on. The specific positioning of these cells gives rise to the perivascular niche, reticular niche and osteoblastic niche, respectively (Fig. 1). Furthermore, some capillary endothelial cells and sinus endothelial cells also express CXCL12, although its expression level is rather weak in normal conditions. CXCL12‐positive cells are located in close proximity to CD34‐positive/c‐kit‐positive hematopoietic cells.^16^ Notably, aging was shown to influence the distribution of CXCL12‐positive cells in the bone marrow, leading to an increase in both cell number and messenger RNA (mRNA) expression level. In contrast, age‐dependent reduction of mRNA expression level of CXCR4 was observed in the bone marrow. The CXCR4 molecule is known to be expressed in the hematopoietic cells/leukemic blasts in the bone marrow.^16^, ^18^ These results might suggest that niche cells play a compensatory and supportive role in hematopoiesis via the CXCL12/CXCR4 axis, thereby mitigating the age‐induced decrease in the hematopoietic potential of hematopoietic cells.^22^

### Apoptotic machinery is specifically upregulated in MDS bone marrow

Studies have shown a significant increase in the number of CD68‐positive macrophages in MDS bone marrow when compared with the number of cells in control samples. Even in the bone marrow of MDS cases that later transformed to overt leukemia (MDS → OL cases, AML with myelodysplasia related changes, AML‐MRC), CD68‐positive cells were significantly more frequent than in control samples. Control samples were consisted with bone marrow samples from cases without hematological disorders. In contrast, CD68‐positive cells were rarely found in the bone marrow of patients with AML.^23^ Pathological examination revealed that the expression of TNF receptor‐I and FAS was upregulated in hematopoietic cells while Tumor necrosis factor alpha (TNFα) and FAS‐L were found to be prominently overexpressed in MDS bone marrow macrophages. These results suggest that apoptotic signals are upregulated in MDS bone marrow via TNFR‐I‐TNFα, FAS‐FASL and other systems.^24^, ^25^, ^26^, ^27^ Moreover, CCL3, TGF‐β, S100A9, S100A8 and interleukin (IL)1β have been shown to be overexpressed in bone marrow stromal cells of patients with MDS, further contributing to the induction of apoptosis in hematopoietic cells.^28^

### Characteristics of MDS bone marrow niches

**Figure 2:**
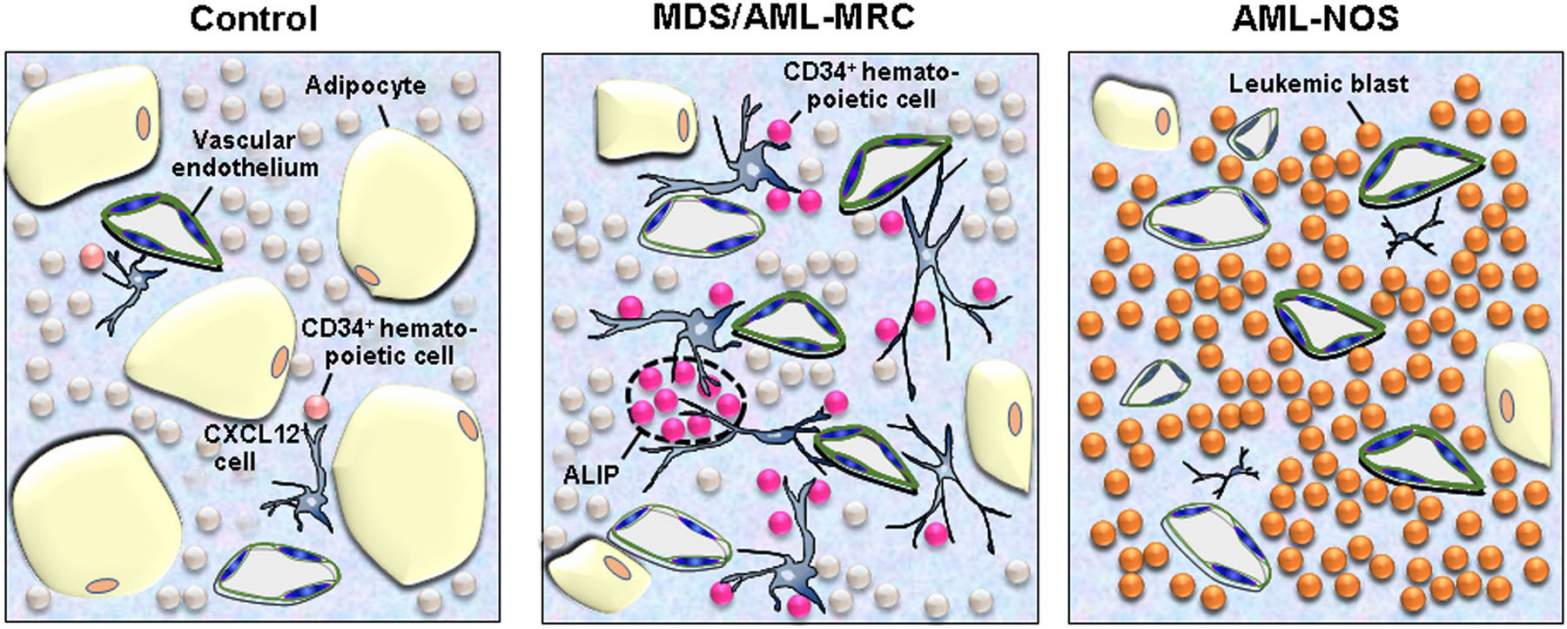
Schematic illustration of the hematopoietic stem cell niche created by CXCL12‐positive stromal cells and hematopoietic cells in the human bone marrow. **Left**: In control bone marrow, CXCL12‐positive cells are scattered in the parenchyma and perivascular area and in contact with CD34‐positive hematopoietic cells, including hematopoietic stem cells. **Middle**: In MDS/AML‐MRC bone marrow, CXCL12‐positive cell density and the number of CD34‐positive hematopoietic cells are both increased. CXCL12‐positive cells are located in the cellular marrow and perivascular area, while the majority of CD34‐positive hematopoietic cells are in intimate contact with CXCL12‐positive cells, as was the case in the control bone marrow. The clusters of CD34‐positive hematopoietic cells (abnormal localization of immature precursors: ALIP) also exhibited close localization with CXCL12‐positive cells. **Right**: In contrast, AML‐NOS bone marrow exhibited decreased CXCL12‐positive cell density whereas leukemia blasts markedly increased throughout the marrow parenchyma.

The expression of CXCL12 was shown to be significantly upregulated in MDS bone marrow when compared with the bone marrow of control samples. Moreover, the number of CXCL12‐positive cells increased and the expression level of CXCL12 mRNA was very high in the bone marrow of patients with MDS. In contrast, CXCL12‐positive cells were only rarely observed in the bone marrow of patients with AML.^29^ A schematic illustration of CXCL12‐positive niche cells in the bone marrow of control, MDS and AML cases were represented in Fig. 2 and our previous report.^29^ Studies from other groups have shown that exosomes derived from AML cells promote the expression of DKK1 in niche cells, resulting in reduced expression of CXCL12, KITL, and IGF‐1 in the bone marrow.^30^ As a result, AML cells modify the behavior of niche cells to an AML‐prone nature, thereby suppressing normal hematopoiesis and inducing the dominant proliferation of AML cells. Thus, the bone marrow microenvironments of MDS → OL (AML‐MRC) and *de novo* AML were found to be completely different from the normal functions of niche cells.^31^ In MDS bone marrow, CD34‐positive/c‐kit‐positive cells are generally located in proximity to CXCL12‐positive cells and express the BCL‐2 protein, resulting in the suppression of apoptosis. As such, niche cells might support the survival of CD34‐positive blastic/immature cells via signal interactions. *In vitro* experiments also revealed that CXCL12 mediated the survival signals of CXCR4‐positive hematopoietic cells via interactions (co‐culture) with CXCL12‐transduced fibroblastic cells through the CXCL12/CXCR4 axis.^29^

Neovascularization in the bone marrow microenvironment would also be an important feature involved in the control of hematopoiesis. The bone marrow microvascular density (MVD) was shown to be significantly higher in MDS/AML cases than in control samples.^32^ However, MVD in MDS → OL (AML‐MRC) cases was significantly lower than when compared to patients with AML. Notably, MVD dynamics seemed different in AML‐MRC and *de novo* AML bone marrow.

### Bone marrow niche cells in myeloproliferative neoplasms

**Figure 3:**
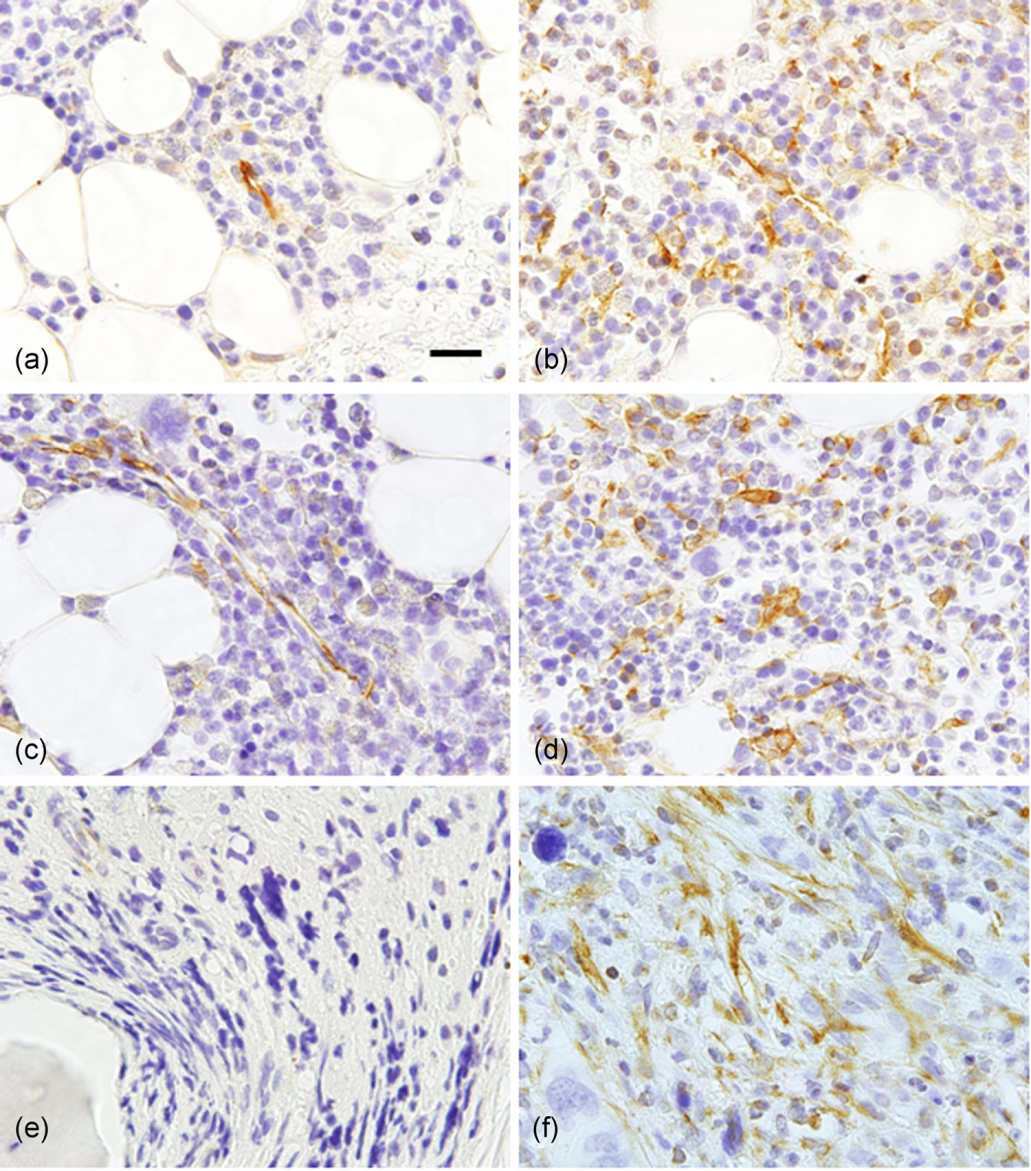
Immunohistochemical localization of CXCL12‐positive cells in the bone marrow of PV (**a,b**), ET (**c**,**d**) and MF (**e,f**). (**a**) PV case with *JAK2* mutation; (**b**) PV case without oncogenic driver mutations (ODM); (**c**) ET case with *CALR* mutation; (**d**) ET case without ODM; (**e**) primary MF with *JAK2* mutation; (f) secondary MF case derived from PV. CXCL12‐positive cells are shown by brown color. Bar indicates 50 μm. Note that only a few CXCL12‐positive vascular endothelial cells existed in cases with ODM (**a,c**) in contrast to moderate to marked increase in number of CXCL12‐positive cells in ODM‐negative cases (**b,d**) and secondary MF (**f**).

**Figure 4:**
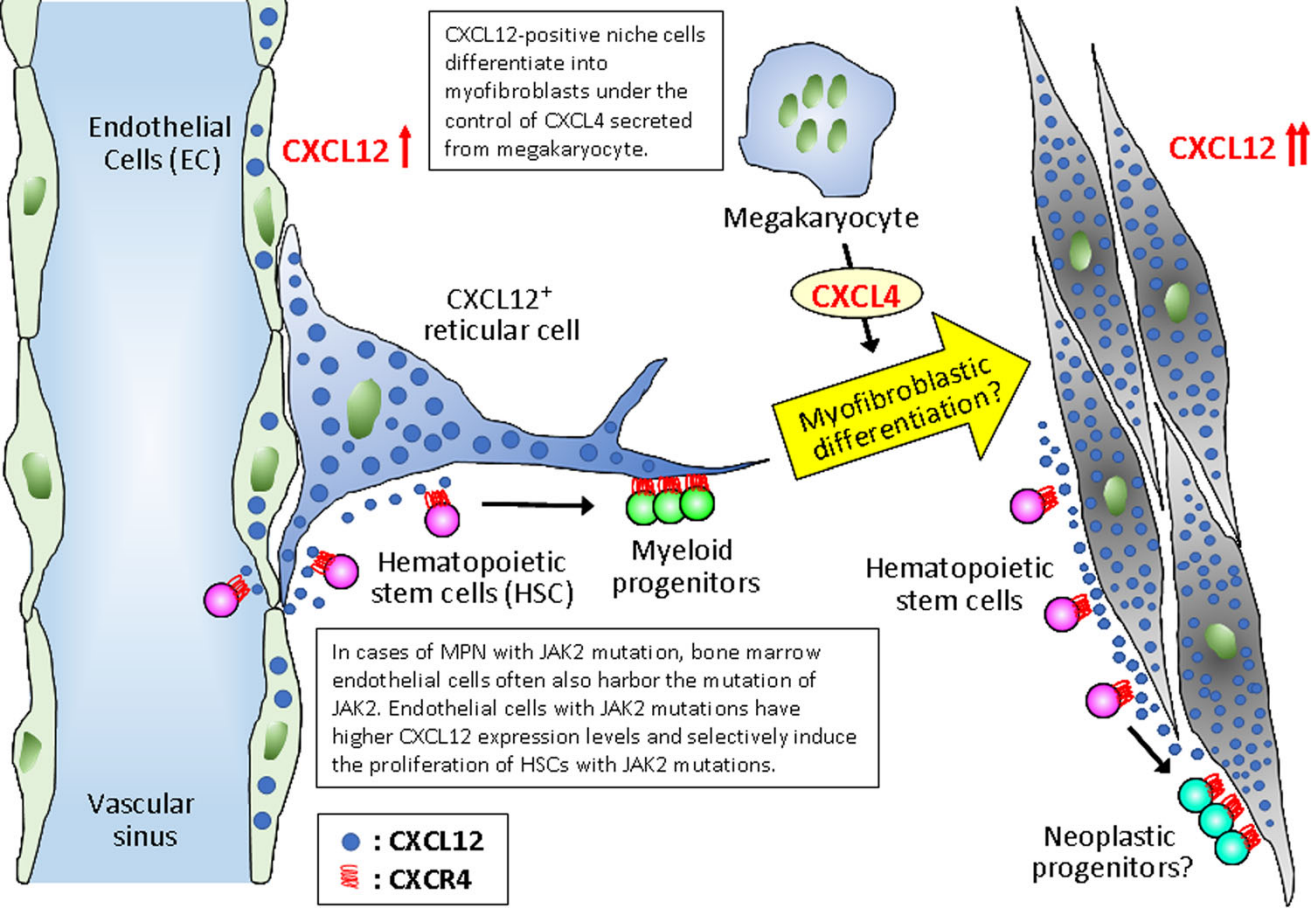
Schematic illustration of the assumed mechanisms of secondary myelofibrosis onset deriving from myeloproliferative neoplasms (MPN) with positive oncogenic driver mutations (ODM). CXCL12‐positive cells in ODM‐positive MPN cases are known to harbor ODM mutations.^35^ These cells might express higher levels of CXCL12 and thus be stimulated to proliferate by megakaryocytes via secretion of CXCL4.^36^

In the bone marrow of CML patients, patients with CML‐CP (chronic phase) exhibited a significantly lower expression of CXCL12 than control samples. However, CXCL12 levels were similar in patients with CML‐BC (blastic crisis) and control samples.^33^ Using mice models with the *BCR‐ABL* transgene, it was shown that the exosome‐derived miR‐126 secreted by CML cells reduced the expression of CXCL12 in bone marrow endothelial cells.^34^ The decreased expression of CXCL12 in bone marrow endothelial cells may induce the outflow of CML cells into the bloodstream due to the suppression of the CXCL12/CXCR4 axis signal, followed by the induction of splenomegaly. Moreover, patients with polycythemia vera (PV), essential thrombocythemia (ET), and primary myelofibrosis (pMF) exhibited characteristic localization patterns of CXCL12‐positive cells in the bone marrow (Fig. 3). Cases with oncogenic driver mutations (ODM), such as Janus kinase 2 (*JAK2)*, calreticulin (*CALR),* and *MPL* mutations, exhibited a prominent decrease in the number of CXCL12‐positive cells in the bone marrow (Table 1). It was previously reported that hematopoietic cells with *JAK2* mutations secreted IL‐1β and induced the destruction of the bone marrow niche via the reduced expression of CXCL12.^28^ In contrast, bone marrow CXCL12‐positive cells were increased in MPN cases without ODM, especially in cases in which PV or ET transformed to secondary MF. The increased number of CXCL12‐positive cells in secondary MF partially expressed vimentin, smooth muscle actin, and CD146, suggesting that these cells underwent myofibroblastic differentiation.^35^, ^36^ Figure 4 shows the schematic illustration of the expected relation of hematopoietic cells and CXCL12‐positive cells in the bone marrow of patients with secondary MF. CXCL12‐positive cells present in these pathologies might harbor ODM,^35^ and thereby could differentiate to myofibroblastic cells.^36^

**Table 1:** Characteristics of the distribution of CXCL12‐positive cells in the bone marrow of MPN

Types of MPN	CXCL12‐positive reticular cells	CXCL12 expression in the endothelial cells of vessels/sinuses
CML‐CP	Rare	Not identified
CML‐BC	More frequent	Not identified
	than CML‐CP	
Polycythemia vera		
ODM‐positive cases	Rare	Upregulated
ODM‐negative cases	Increased	Upregulated
Essential thrombocythemia		
ODM‐positive cases	Rare	Upregulated
ODM‐negative cases	Increased	Upregulated
Myelofibrosis		
Primary MF (pMF)	Rare	Upregulated
Secondary MF derived from other MPN	Markedly increased in the spindle‐shaped cells	Upregulated

Abbreviations: MPN, myeloproliferative neoplasms; ODM, oncogenic driver mutations (JAK2, CALR, MPL etc.).

## PATHOLOGY OF MYELODYSPLASTIC SYNDROMES (MDS)

### Regulation of cell proliferation is a key step of the transformation of myelodysplastic syndromes to overt leukemia

In the bone marrow of patients with myelodysplastic syndromes (MDS), hematopoietic cells are characterized by increased cellular proliferation as well as frequent cell death induced by apoptotic processes.^13^, ^14^ When the initial events of MDS onset occur, MDS clone cells should be characterized by increased proliferative activity rather than apoptotic features, as the MDS clone may not be able to be sustained if apoptotic processes are dominant.^15^ However, it would be very difficult to detect the initial phase of MDS onset before the clinical manifestations become apparent. Therefore, in order to elucidate the proliferative character of MDS clone cells, we preferred to analyze the events that occur during the transformation of MDS to overt leukemia (OL), in which cell proliferation processes will be strongly enhanced. One of the most important genetic events that occur in this process is the mutation of the *p53* gene, which is the representative mechanism for avoiding apoptosis employed by MDS clone cells. In the bone marrow of MDS patients, 20% of patients were found to be p53 positive by immunohistochemical analysis.^24^

In p53 positive MDS cases, p53 positivity could be identified in an earlier phase of the disease, namely refractory anemia (RA), and the transformation to OL could be exclusively observed in these p53‐positive RA cases.^24^ Therefore, p53 abnormalities might serve as a driver for the transformation of MDS to OL, rather than an initial step of MDS onset. Similarly to the p53 status, abnormalities in DNA‐damage checkpoint molecules such as ATM, Chk2, and H2AX might occur during the transformation of MDS to OL.^37^ Moreover, studies have revealed a decreased expression of miR‐29b, resulting in the activation of the anti‐apoptotic protein MCL‐L1 and the decrease in the number of apoptotic cells during the transformation of MDS to OL.^38^ Furthermore, CIP2A was also shown to have an increased expression, resulting in the inhibition of PP2A, excess phosphorylation of c‐Myc and thus, the activation of cellular proliferation.^39^ Overexpression of CIP2A was selectively observed in myeloid series of hematopoietic cells and CD34‐positive immature cells in MDS bone marrow. These findings are consistent with the fact that the proliferation of the myeloid series of cells occurs during the transformation of MDS to OL. The genome‐wide and whole genome analyses of MDS/OL cases have revealed the additional gene mutations during progression from MDS to OL such as *TP53*, *GATA2*, *KRAS*, *RUNX1*, *STAG2*, *ASXL1*, *ZRSR2* and *TET2* mutations (low risk MDS to high risk MDS), and *FLT3*, *PTPN11*, *WT1*, *IDH1*, *NPM1*, *IDH2* and *NRAS* mutations (MDS to OL).^40^, ^41^

### Host genetic factors control the apoptotic frequency of hematopoietic cells in MDS bone marrow

Which molecular mechanisms would influence the frequency of the apoptotic process in hematopoietic cells, in association with the other characteristic features of MDS in the bone marro? Through analyses of retrovirus‐induced leukemia in animal models, we could identify the mice strain C3H, that had hematopoietic cells with an apoptosis‐prone nature after infection with Friend leukemia virus (FLV) and DNA‐damage.^42^, ^43^ A genome‐wide analysis demonstrated that the enhanced expression of the minichromosome maintenance 2 (MCM2) protein played a key role in the process of strain‐specific induction of frequent apoptosis in the bone marrow hematopoietic cells of C3H mice.^44^ The MCM2 protein primarily serves as a helicase during DNA‐replication by forming the MCM complex with other MCM family proteins in normal cells/tissues. In specific hosts with high MCM2 expression levels which are genetically‐induced, such as C3H mice, hematopoietic cells show an apoptosis‐prone nature when the combination of viral infection and DNA‐damage occur.^44^, ^45^ Furthermore, these findings could also be used for therapies in which tumor cell apoptosis could be induced in tumors expressing high levels of MCM2, such as triple‐negative breast cancers and a fraction of ovarian cancers.^45^

How does MCM2 expression influence the induction of apoptosis in hematopoietic cells of the human bone marrow? Although similar genetic mutations frequently occur in patients with AML and MDS, MDS cells show frequent apoptosis while AML cells do not necessarily exhibit apoptosis. As such, we evaluated MCM2 expression levels as a possible cause of the differences in hematopoietic cell dynamics of AML and MDS. As described above, MCM2 is essential in the process of DNA‐replication. Therefore, highly proliferative tumor cells usually exhibit high MCM2 expression levels. Likewise, we could observe a significant positive correlation between the expression levels of MCM2 and the Ki‐67‐positive cell ratio in the bone marrow of control samples and AML cases. However, in the bone marrow of MDS patients, MCM2 expression and Ki‐67 ratio were not found to be correlated. Instead, MCM2 expression levels showed a positive correlation with the cleaved caspase‐3‐positive apoptotic cell ratio.^46^ *In vitro* experiments using MDS‐derived hematopoietic cell lines demonstrated that MCM2 overexpression induced apoptosis in MDS hematopoietic cells.^46^ Thus, the function of MCM2 was completely different between hematopoietic cells from patients with MDS and those from control bone marrow/AML cells. The expression level of MCM2 was significantly higher in the bone marrow of MDS patients than in the bone marrow of control samples and AML cases. Thus, higher expression of MCM2 in the hematopoietic cells and higher expression of CXCL12 in the niche cells would contribute to form the characteristic host status of MDS bone marrow.

**Figure 5:**
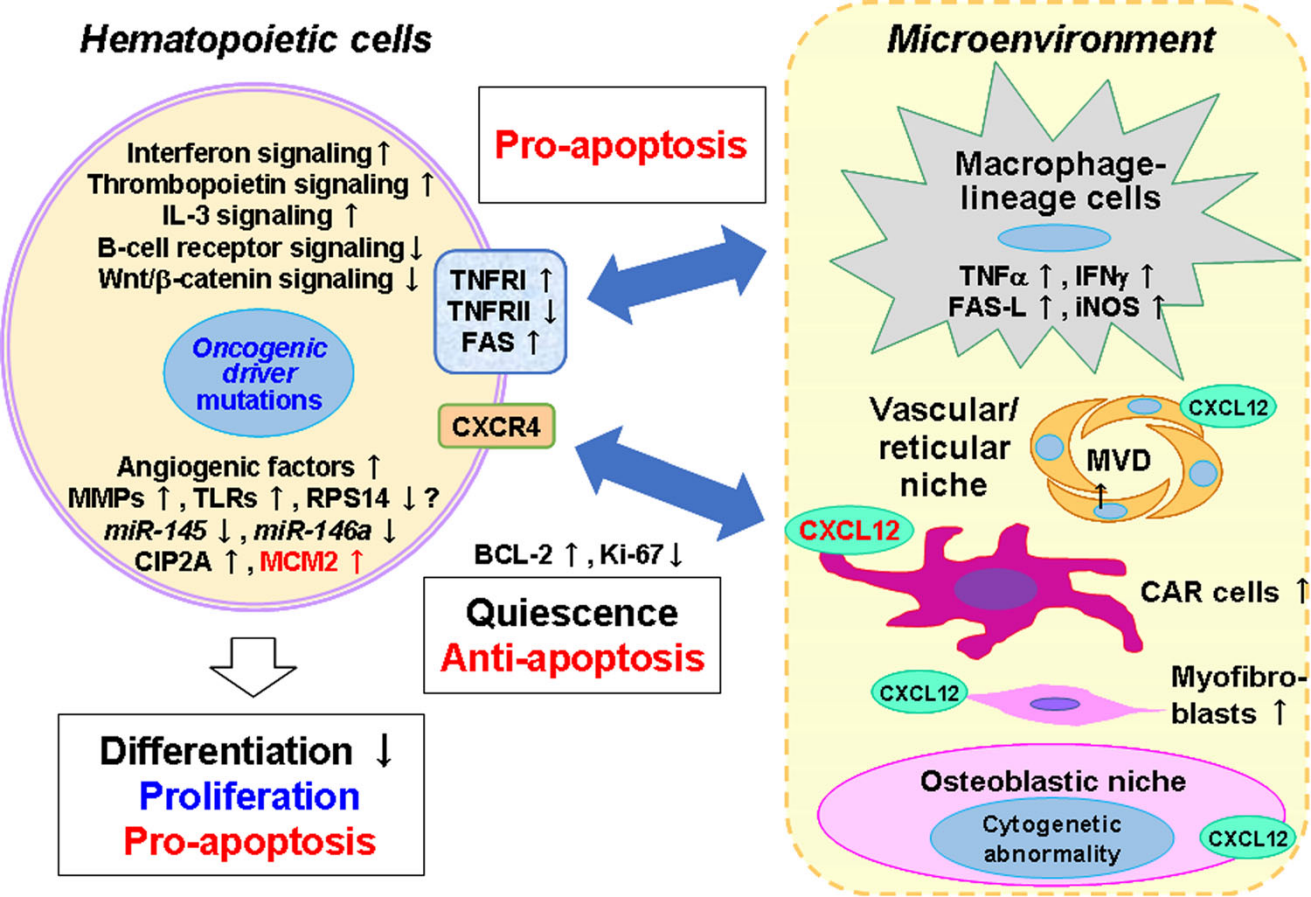
Abnormalities identified in myeloid neoplasms (MDS). Abnormal phenotypes of MDS such as bone marrow cell proliferation/differentiation as well as apoptosis of hematopoietic cells occur through the interaction between hematopoietic cells and the cells present in the microenvironment. Among the cells in the microenvironment, macrophage‐lineage cells offer pro‐apoptotic signals to hematopoietic cells, in contrast to niche cells, which seem to regulate the antiapoptotic stimuli of hematopoietic cells in the bone marrow.

The expression levels of molecules associated with proliferative/apoptotic signals in MDS bone marrow are summarized in Fig. 5. Interactions between hematopoietic cells and mesenchymal (stromal) cells, including macrophages and CXCL12‐positive cells, in the bone marrow, might have a very important role in generating the MDS pathology.

## CONCLUSION

The fact that clonal hematopoiesis of indeterminate potential (CHIP) or clonal cytopenia of unknown significance (CCUS) exists among cases associated with MDS suggests that the cause of MDS and myeloid neoplasms might be related to not only genetic mutations of hematopoietic cells but also to host background factors controlling the condition of hematopoietic cells themselves and the bone marrow microenvironment/niche. As mentioned in the present manuscript, hosts with higher expression of MCM2 might be more susceptible to MDS‐type myeloid neoplasms while MCM2‐low hosts have a higher risk of developing AML or MPN when genetic mutations occur in hematopoietic stem cells. Furthermore, genetic background, genetic mutations, and interaction of hematopoietic stem cells/niche cells would influence the type of myeloid neoplasms developed. The interactions of hematopoietic stem cells and niche cells have been clarified to regulate the normal hematopoiesis through cell proliferation/differentiation in the bone marrow.^16^ By contrast, niche cells in AML interfere with the normal hematopoiesis but support the proliferation of leukemic blasts.^18^, ^21^ In MDS bone marrow, niche cells showed marked increase in number and supported the survival of hematopoietic cells. The hematopoietic cells/niche cells interactions of MPN cases were rather complicated. The functions of niche cells of MPN cases appeared different between cases with ODM and without ODM. As such, clarifying these interactive factors may hopefully lead to the development of novel therapeutic targets and approaches against myeloid neoplasms.

## DISCLOSURE STATEMENT

None declared.

## AUTHOR CONTRIBUTIONS

MKi contributed to the conception and design of the study, acquire and analyzed data and drafted the manuscript, table and figures. MKu, IO and KY acquired and analyzed data and discussed about the interpretation of data.
